# Long-term survival in an acute promyelocytic leukemia patient with recurrent granulocytic sarcomas: A case report

**DOI:** 10.1097/MD.0000000000025257

**Published:** 2021-04-09

**Authors:** Xuehui Zhou, Chengwen Li

**Affiliations:** a Department of Hematology, PLA Strategic Support Force Medical Center, Beijing; b Department of Pathology, Institute of Hematology and Blood Diseases Hospital, Chinese Academy of Medical Sciences & Peking Union Medical College, Tianjin, China.

**Keywords:** acute promyelocytic leukemia, diagnosis, extramedullary disease, recurrent granulocytic sarcoma, treatment

## Abstract

**Rationale:**

Granulocytic sarcoma (GS) is an extramedullary myeloid tumor composed of immature cells of the granulocytic series. It rarely occurs in acute promyelocytic leukemia (APL). No case of long-term survival in an APL patient with recurrent GS has been reported.

**Patient concerns:**

A 54-year-old female patient was diagnosed with APL in 1995 and has been in complete remission (CR) of bone marrow morphology for 24 years; however, recurrent GS occurred successively in ovary, breast, spine, body of sternum, lymph nodes, soft tissues from 2004 to 2019.

**Diagnoses:**

The immunohistochemistry confirmed the diagnosis of GS, and fluorescence in situ hybridization (FISH) revealed its origin from APL.

**Interventions:**

She received surgery, and had an excellent response to all-trans retinoic acid (ATRA), DA (daunorubicin combined with cytarabine) regimens, and arsenic trioxide (ATO).

**Outcomes:**

The patient achieved CR in March 2020 after radiotherapy followed by ATO and ATRA. So far, she is still in follow-up.

**Lessons:**

It is rare that recurrent GS at multiple sites is involved in APL patient with bone marrow morphology in CR. It is interesting to observe a long-term excellent response to ATRA, chemotherapy and ATO. Although multiple recurrence of GS in patients with APL is rare, the data in this case highlight the need for individualized treatment when such conditions occur.

## 1 Introduction

Granulocytic sarcoma (GS) can occur in patients with acute myeloid leukemia (AML), myelodysplastic syndrome, or chronic myelogenous leukemia. It is usually found concomitantly with or after the onset of AML.^[1]^ The incidence of GS in AML varies from 2% to 10.4%, and most of the cases are associated with AML FAB M2, M4 and M5 while less with M3 (namely APL) (2.2%, 1/45).^[2]^ Most relapse of extramedullary APL tends to occur in the central nervous system (CNS).^[3]^ The most common sites of GS are the skin, orbit, bone, periosteum, soft tissue, and lymph nodes. Other organs involved, including the breast, prostate, urinary bladder, pericardium, ovary, pancreas, and the uterus, have also been reported in the literature.^[1–3]^ The range of patient age is from 2 to 81 years (mean 48).^[1]^ Here, a case of recurrent GS in a patient with APL is presented.

## 2 Case presentation

**Figure 1:**
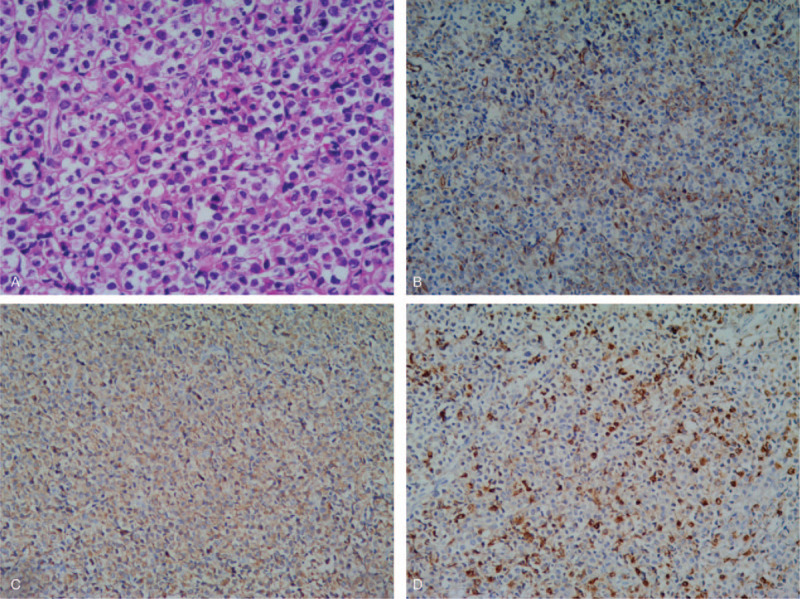
Pathological results of breast in 2004, supporting GS, (A) H&E staining, (B) immunohistochemistry staining positive for CD34, (C) cytoplasmic expression of MPO, (D) cytoplasmic expression of Lysozyme; Magnification: A×200; B, C, D×100.

**Figure 2:**
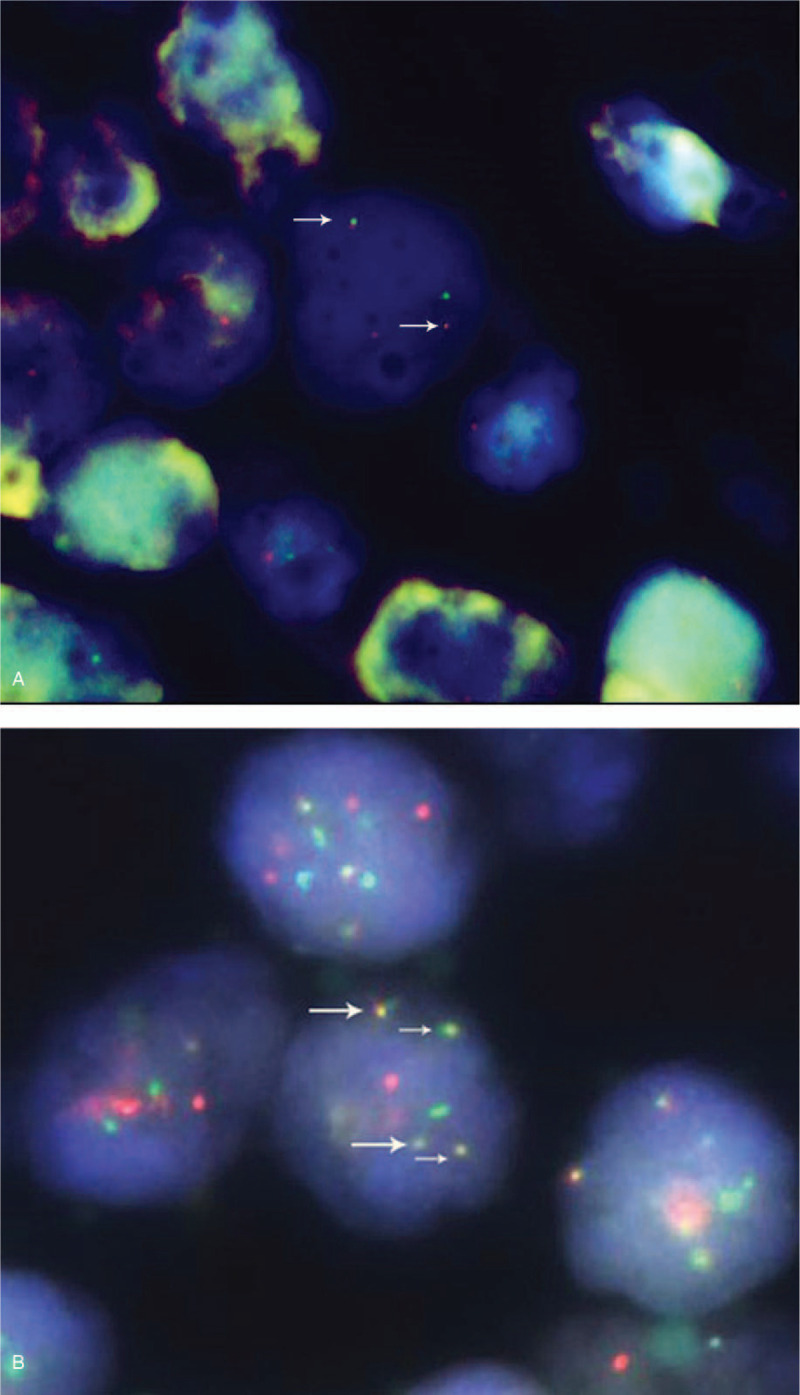
FISH analysis done on cells derived from breast tissue in 2004 (A) and lymph node tissue in 2015 (B), red (PML), green (RARA), and 2 fusion (PML/RARA) signal pattern is observed.

On August 16, 2004, a 40-year-old female farmer came to our hospital complaining of numbness in the legs and a hard mass of 3 cm diameter in the upper outer quadrant of the right breast for 2  weeks. In May 1995, her leukocyte on presentation was 1.4 x 10^9/L; hemoglobin, 114 g/L; and platelets, 0 x 10^9/L, then she received ATRA (45 mg/m^2^/day, 4 weeks on 4 weeks off, for a total of 6 cycles) for APL and achieved complete remission (CR) of bone marrow morphology. In May 2004, she underwent excision of right ovary, which was pathologically diagnosed as lymphoma at other hospital and reviewed as GS by us in August 2004. In July 2004, she underwent excision of a mass in the lower outer quadrant of the right breast at another hospital, where the pathological diagnosis was unclear. The patient has no family history and no occupational radiation exposures. On physical examination, under the ninth thoracic level, hypoesthesia and paraparesis were present, deep tendon reflexes were hyperactive, and the Babinski test was positive bilaterally. Magnetic resonance imaging (MRI) revealed a solid lesion that was intraspinal extramedullary located between the ninth and tenth thoracic vertebral levels. Peripheral blood smear and bone marrow examinations were normal. The total laminectomy procedure at thoracic 9 to 10 levels and lumpectomy of breast were performed, and the pathological results revealed GS (Fig. 1 Breast, A. H&E staining, B. CD34+, C. MPO+, D. Lysozyme+; magnification: A×200; B, C, D×100). PML/RARA fusion signal by FISH was positive for breast [Fig. 2A, red (PML), green (RARA), arrow (PML/RARA), reviewed in 2015]. There were no symptoms on the fifth day after the operation. She received DA regimen (daunorubicin 45 mg/m^2^/day, d1∼3; cytarabine 150 mg/m^2^/day, d1∼7, 2 cycles) and ATRA (25 mg/m^2^/day, d1–28, 4 cycles).

In January 2011 and April 2012, the patient received DA regimen (1 cycle and 2 cycles, respectively) and ATRA (25 mg/m^2^/day, d1-28, 2 cycles) due to unexplained intermittent chest pain, and the symptoms disappeared, respectively. Despite CR of bone marrow, it was speculated that chest pain was closely related to APL.

**Figure 3:**
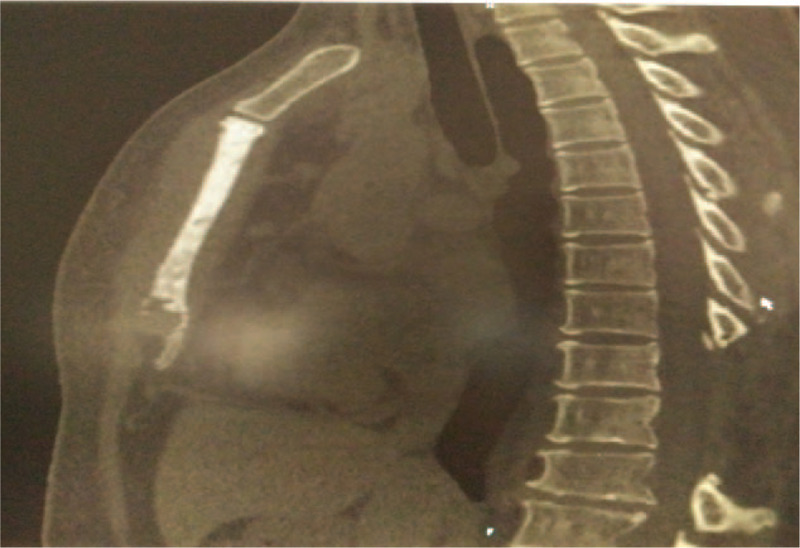
Chest CT revealed the increase of bone density in the body of sternum on February 5, 2015.

In January 2015, she presented with a 3-month history of worsening chest pain and rapid bilateral axillary lymph node enlargement. On examination, there was a lymph node of 2 cm diameter on each side of the armpit. On the basis of biopsy result, left axillary lymph node was GS. The immunohistochemistry examination showed bcl-2(+), CD34(+), CD117(+), MPO(+), Lysonzyme(+), TDT(+), Ki67(+5∼10%), CD3(−), CD5(−), CD20(−), P53(−), CD10(−), CD68(−), CD30(−), CD(79), Bcl-6(−), CD21(−), CD56 (−), CD15 (−), ALK (−), CyclinD1 (−), Mum-1 (−), CD23 (−), CD38(−). Peripheral blood smear, bone marrow examinations, and flow cytometry of bone marrow were normal. Conventional cytogenetics of bone marrow revealed a normal karyotype of 46, XX [20], but PML/RARA fusion gene by reverse transcription-polymerase chain reaction (RT-PCR) was 0.019% (14/73403, long-form). In addition, PML/RARA fusion signal by FISH was positive for lymph node (Fig. 2B). Computed tomography (CT) scan of chest revealed the increase of bone density in the body of sternum (Fig. 3). She received DA regimen (2 cycles) after which no enlarged lymph nodes were found by ultrasonography, and then received another DA, EA (etoposide70 mg/m^2^/day, d1∼4; cytarabine 150 mg/m^2^/day, d1∼7) regimens, and ATRA (25 mg/m^2^/day, d1∼28, 2 cycles). She received prophylactic intrathecal chemotherapy (dexamethasone 10 mg, cytarabine 30 mg, methotrexate 15 mg) during induction and consolidation therapy.

In October 2017, she presented with a lump in chest for 2 months, of size 4.6 × 4.1 × 1.5 cm by ultrasonography. The ultrasound-guided mass fine needle aspiration biopsy was performed. The immunohistochemistry examination showed CgA(−), Syn(−), bcl-2(+), CD68(−), CD99(+), Ki67(+15∼20%), LCA(+), CD117(−), CD56(−), MPO(+), TdT(−), Lysozyme(−). PML/RARA fusion signal by FISH was positive for the mass. Morphology and cytogenetics were normal, but PML/RARA fusion gene was 0.011% (5/47,237). So, she received MA (mitoxantrone 8 mg/m^2^/day, d1∼3; cytarabine 150 mg/m^2^/day, d1∼7) regimen for 1 cycle, and subsequently received ATO (0.15 mg/kg/day, d1∼28), ATRA (25 mg/m^2^/day, d1∼28) and ATO (0.15 mg/kg/day, d1∼28). No mass in chest was found by ultrasonography in January 2018.

**Figure 4:**
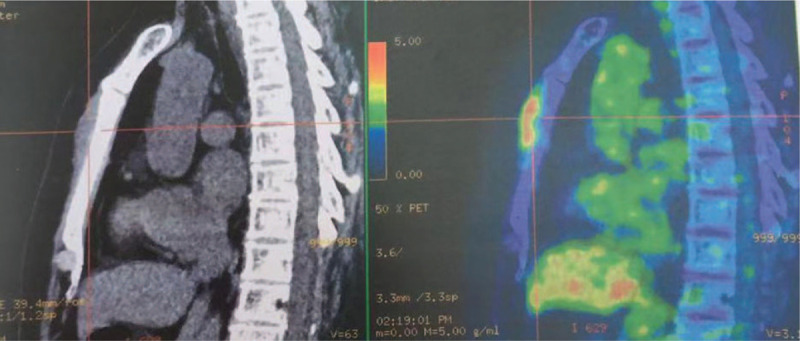
18FDG-PET-CT revealed a mass anterior to the body of the sternum and FDG uptake in the lesion, with a maximal standardize uptake value (SUVmax) of 5.7 on October 28, 2019.

In October 2019, she presented with intermittent chest pain for 1 month. Morphology, cytogenetics, and molecular genetics were normal. Systemic examination by 18F–fluorodeoxyglucose-positron emission tomography-computed tomography (18FDG-PET-CT) revealed a mass anterior to the body of the sternum and FDG uptake in the lesion, with a maximal standardize uptake value (SUVmax) of 5.7 (Fig. 4). Biopsy was not performed due to high risk. Because of the history of recurrent promyelocytic sarcomas, GS originating from APL was speculated. She received radiotherapy of total 50Gy in 25 times on sternal bone with anterior soft tissue followed by ATO (0.15 mg/kg/day, d1∼14, 2 cycles) and ATRA (25 mg/m^2^/day, d1∼14, 4 cycles), and no mass anterior to the sternum was found by ultrasonography in March 2020.

## 3 Discussion

GS is an extramedullary myeloid tumor composed of immature cells of the granulocytic series. It can occur in any organ or tissue. Within leukemic APL patients, GS is rare and mostly occurs in the relapse phase, and also earlier or accompanied with APL.^[4]^ High leukocyte count and younger age are suggested as risk factors of extramedullary APL.^[5,6]^ The durations from diagnosis of bone marrow disease to development of GS ranged from 11 to 50 months in APL,^[7]^ but in the report by us, the duration was 141 months. Isolated GS is rare, and its clinical features may differ from extramedullary disease (EMD) in advanced APL. Many cases occur as a spinal tumor, and some occur in the absence of bone marrow diseases.^[4]^ Isolated GS is frequently mistaken for lymphoma, small round cell tumor, or undifferentiated carcinoma, especially when immunohistochemistry is not used.^[8]^ FISH analysis of GS tissue is useful for accurate diagnosis, even in preserved tissue. Because EMD usually occurs in the relapse phase and many CNS cases are included, survival after developing EMD is poor.^[4]^ A retrospective review showed that failure to achieve CR with induction therapy, and age >65 years are associated with poor outcomes in GS.^[9]^

According to NCCN guidelines in 2021, induction therapy with anthracycline-based regimen or ATO + ATRA ± a single dose of gemtuzumab ozogamicin or anthracycline is recommended for relapse APL according to recurrence time and initial treatment plan. If CR is not achieved, hematopoietic stem cell transplantation (HSCT) or clinical trials are recommended. Recurrent GS of APL needs a combination of ATRA, ATO, chemotherapy, radiotherapy, surgery and HSCT. Harris et al^[10]^ reported a case in which a 25-year-old man with APL underwent multiple relapses of GS at multiple anatomical sites (left scapula, thoracic vertebra, right atrium, and supraclavicular mass). Many methods, including ATRA, idarubicin, doxorubicin, gemtuzumab ozogamicin, ATO, local radiation, allogeneic HSCT, and surgery were invalid, and tamibarotene helped the patient obtain CR for 12 months. The treatment of both GS and APL is still inconclusive. A study of relapsed APL showed that the actuarial overall survival (OS) for the 3 protocols leveled off by 2 years at 82% for ATO and ATRA, 43% for bone marrow transplantation, and 23% for chemotherapy.^[11]^ Furthermore, for patients with relapsed APL, ATO remained effective despite repeated ATO exposures.^[12]^

No long-term survival in an APL patient with recurrent GS was reported in the past. The patient reported by us received ATRA for APL in 1995, which was verified to be effective, and was not taken in combination with chemotherapy due to her financial difficulties. After recurrent GS in 2004, she was repeatedly hospitalized for EMDs involving ovary, breast, spine, body of sternum, lymph nodes, and soft tissues, but has been in CR of bone marrow morphology for 24 years. Because of economic reasons, no molecular biology detection was done from 2004 to 2012. Retrospective analysis of the preserved initial GS tissue sample revealed PML/RARA fusion gene by FISH, conforming the diagnosis of the GS type of APL. In 2015 and in 2017, she had a molecular relapse. Later, she was diagnosed as isolated GS in 2019. She received effective and economical therapy with surgical operation, ATRA, chemotherapy, ATO and radiotherapy. So, it is a very unusual case. The reason for long-term survival may be attributed to the late relapse which is confined to be of extramedullary nature only. In addition, medication as less as possible is also an important factor. For low-risk patients of APL, nonexcessive treatment may be beneficial.

ATRA and ATO have greatly modified the prognosis of APL. APL is now considered a curable disease. CR of APL has reached 94.3%, The event-free survival estimate at 12 years was 68.9%.^[13]^ The frequency of extramedullary relapse in APL is thought to be associated with the introduction of ATRA as a component of APL therapy. ATRA-driven differentiation process induces alteration in expression of the cell surface adhesion molecules, which could facilitate infiltration of leukemic cells into extramedullary sites.^[14]^ However, in 2001, a study showed that ATRA did not increase the incidence of extramedullary recurrence of APL.^[15]^ Although CD56 expression is an indicator of poor clinical outcome in patients with APL treated with simultaneous all-trans retinoic acid and chemotherapy,^[16]^ no correlation was found with the cellular adhesion molecule CD56 in this case.

In conclusion, the case in APL is rare, in which recurrent GS at multiple sites is involved and bone marrow morphology is continuously CR. It is interesting to have such a long-term excellent response to ATRA, chemotherapy, and ATO. Although multiple recurrence of GS in patients with APL is rare, the data in this case highlight the need for individualized treatment when such conditions occur.

## Acknowledgments

The authors thank Dr. Yunge Gao, Shusen Zhai and Tonghuan Zhen of oncology department of PLA strategic support force medical center, for their treatment work in the case reported. The authors thank the patient and her family for providing informed consent for the publication of this case report.

## Author contributions

**Conceptualization:** xuehui zhou.

**Formal analysis:** xuehui zhou.

**Methodology:** chengwen li.

**Writing – original draft:** xuehui zhou.

**Writing – review & editing:** xuehui zhou.
